# An Adaptive Fuzzy-Logic Traffic Control System in Conditions of Saturated Transport Stream

**DOI:** 10.1155/2016/6719459

**Published:** 2016-07-19

**Authors:** N. R. Yusupbekov, A. R. Marakhimov, H. Z. Igamberdiev, Sh. X. Umarov

**Affiliations:** Department of Information Technology, Tashkent State Technical University, 100095 Tashkent, Uzbekistan

## Abstract

This paper considers the problem of building adaptive fuzzy-logic traffic control systems (AFLTCS) to deal with information fuzziness and uncertainty in case of heavy traffic streams. Methods of formal description of traffic control on the crossroads based on fuzzy sets and fuzzy logic are proposed. This paper also provides efficient algorithms for implementing AFLTCS and develops the appropriate simulation models to test the efficiency of suggested approach.

## 1. Introduction

One of the most important and promising tasks of transport systems in modern cities is to satisfy the needs of both state and citizens in efficient transport services. Prior studies investigated methods of improving operational efficiency of transport management systems in different countries [1, 2]. The results demonstrate that the most promising direction is the development and implementation of intelligent transport systems (ITS). Intelligent transportation system (ITS) is an integration of advanced information and communication technologies, computer-aided control and traffic management, transport infrastructure, vehicles, and users, to improve safety and efficiency of road traffic [3].

Today, the scope of the ITS implementation ranges from solving problems of traffic-light management and road safety to increasing the efficiency of the existing transport system. Capabilities of the ITS are not limited to improving the safety and efficiency of the transporting processes. It builds information retrieval systems, focused on the identification of vehicles, the analysis of traffic situations, and detecting violations. Furthermore, ITS allows searching violators, stolen vehicles, and suspected criminals. Such operations are based on remote video monitoring, collection, and intellectual processing of large amounts of data, as well as automatic generation of analytical reports. The reports include both simple and integrated decision-making mechanisms.

The review of the extant ITS projects showed that the latest advances in information and communications technologies, control systems theory, data mining, and analysis techniques are not widely implemented in road traffic management. The reason is the lack of extensive research and efficient methods of synthesis of multilevel intelligent systems in conditions of fuzziness of parameters of the road traffic. There are a number of studies on automating the development of control systems and their components. They are focused on developing and improving the systems of coordinated traffic management on highways and adaptive management that addresses problems of vehicle throughput [2–4]. Furthermore, there are a number of studies that focus on optimizing the traffic-light management [3–8]. A significant number of works are devoted to the development of technical methods for measuring the parameters of road traffic and their processing [4–9]. However, these works are highly scattered and focus on solving local problems of automated control of road traffic. They do not take into account that the improvement of road traffic in a particular area may lead to deterioration of the traffic situation on the other site. Another factor that is not considered by previous studies is that synthesized parameters of traffic-light management systems are optimal only under certain conditions—when the parameters of road traffic do not change over time (or change in short intervals). In most cases, the synthesis of control systems of road traffic does not consider unpredictable traffic situations, fuzziness of the parameters of the road traffic.

Therefore, developing conceptual framework and effective methods of structural and parametric synthesis of control systems of road traffic and development of information technology support systems on their basis is important.

## 2. Statement of a Problem

Below, we consider a class of traffic control systems with imprecise information about the object, in which the qualitative characteristics of the road traffic are dominant.

In general, for synthesis of the traffic control systems, the object of control can be formally represented as follows:

(1) $$\begin{array}{c} M=\langle \;\Omega ,G,X,Y,U,T,\rho ,\gamma ,\zeta \;\rangle , \end{array}$$

where *Ω* = {*Ω*_1_, *Ω*_2_,…, *Ω*_*n*×*m*_} is the range of conditions (e.g., object, output); *G* = 〈*V*, (*D*, *W*)〉 is a model of the crossroad, represented by the graph *G* in the space *ℜ* ^3^; *V* = {*v*_*i*_} are the vertices (nodes) of the graph, corresponding to the nodes of possible branches of the road traffic; *D*⊆*V* × *V* is the plurality of arcs of the graph (road section, connecting nodes of possible branches of road traffic) with corresponding weight coefficients as *W* = 〈Λ, *P*, *Z*〉 (intensity of road traffic, density of road traffic, and average speed on this particular road section); *X* = {*X*_1_, *X*_2_,…, *X*_*n*_} is the plurality of characteristics and determinants, describing the state of the control object Ω and taking their values each in his own set of values {*X*_*i*_}; *Y* = {*Y*_1_, *Y*_2_,…, *Y*_*m*_} is the range of output values (observed processes, parameters, estimates, etc.); *U* = {*U*_1_, *U*_2_,…, *U*_*r*_} is the range of controls (decisions); *T* = {*t*_1_, *t*_2_, …, *t*_*l*_,…} is the time (discrete or continuous); *ρ* : *X* × *U* × *T* → *Ω* is the description of the dynamics of the state of the object, the reaction of the dynamic system in a particular state to control actions; *γ* : *Ω* × *T* → *Y* is the output, describing the observation process of the control object (obtaining estimates, opinions, etc.); *ζ* are some external uncontrollable factors, conditions, and others that have an impact on the dynamics of the control object.

The analysis of transport flows on the crossroads as controlled objects demonstrated that their mathematical model should be able to deal with information fuzziness and describe random values and processes invariantly to their distribution. Mathematical models should also provide math formalism to express expert knowledge, rich empiricism, and heuristics.

According to aforementioned requirements, a generalized dynamic model of the object controls (OC) can be defined as the following linear equation with fuzzy state space [9, 10]:

(2) x−˙=A−⊗x−⊕B−⊗u−,μS−S,S⊆Ω,y−=C−⊗x−,

with fuzzy initial conditions

(3) $$\begin{array}{c} {\overset{-}{x}}_{1}\left(\;0\;\right)={\overset{-}{D}}_{1},\\ {\overset{-}{x}}_{2}\left(\;0\;\right)={\overset{-}{D}}_{2},\\ \vdots \\ {\overset{-}{x}}_{n}\left(\;0\;\right)={\overset{-}{D}}_{n}, \end{array}$$

where ⊗, ⊕ are the fuzzy operations of multiplication and addition; *u* is a control signal (scalar) that accepts discrete numerical value; $\overset{-}{x}=\{{\overset{-}{x}}_{1},{\overset{-}{x}}_{2},\ldots ,{\overset{-}{x}}_{n}\}$ is the state space vector, $\overset{-}{y}=\{{\overset{-}{y}}_{1},{\overset{-}{y}}_{2},\ldots ,{\overset{-}{y}}_{m}\}$ is the output variables vector, $\mu_{\overset{-}{S}}(S)$ is the membership function (MF) of the state space of the object controls, $S=\{s_{1},s_{2},\ldots ,s_{\overset{-}{N}}\}$;

(4) A−=A−11⋯A−n1⋮⋮⋮A−1n⋯A−nn,B−=B−1⋮B−n,C−=C−11⋯C−n1⋮⋮⋮C−11⋯C−n1

are the matrix of the fuzzy coefficients of the model, where

(5) $$\begin{array}{c} {\overset{-}{A}}_{1}^{1}=\left\{\;\frac{A_{1}^{1}}{\mu_{{\overset{-}{A}}_{1}^{1}}\left(A_{1}^{1}\right)}\;\right\},\\ \vdots \\ {\overset{-}{A}}_{n}^{n}=\left\{\;\frac{A_{n}^{n}}{\mu_{{\overset{-}{A}}_{n}^{n}}\left(A_{n}^{n}\right)}\;\right\},\\ {\overset{-}{B}}^{1}=\left\{\;\frac{B^{1}}{\mu_{{\overset{-}{B}}^{1}}\left(B^{1}\right)}\;\right\},\\ \vdots \\ {\overset{-}{B}}^{n}=\left\{\;\frac{B^{n}}{\mu_{{\overset{-}{B}}^{n}}\left(B^{n}\right)}\;\right\},\\ {\overset{-}{C}}_{1}^{1}=\left\{\;\frac{C_{1}^{1}}{\mu_{{\overset{-}{C}}_{1}^{1}}\left(C_{1}^{1}\right)}\;\right\},\\ \vdots \\ {\overset{-}{C}}_{n}^{1}=\left\{\;\frac{C_{n}^{1}}{\mu_{{\overset{-}{C}}_{n}^{n}}\left(C_{n}^{1}\right)}\;\right\}. \end{array}$$

Some *i*th state space vector as a time function *t* can be described as fuzzy relation [1, 3]:

(6) $$\begin{array}{c} {\overset{-}{x}}_{i}\left(\;t\;\right)=\left\{\;t,\frac{x_{i}}{\mu_{{\overset{-}{x}}_{i}}\left(t,x_{i}\right)}\;\right\},\;i=1,2,\ldots ,n. \end{array}$$

At a fixed time, this variable can be expressed as a fuzzy set:

(7) $$\begin{array}{c} {\overset{-}{x}}_{i}=\left\{\;\frac{x_{i}}{\mu_{{\overset{-}{x}}_{i}}\left(x_{i}\right)}\;\right\}. \end{array}$$

A similar description is *j*th output variable:

(8) y−jt=t,yjμy−jt,yj,j=1,2,…,m,y−j=yjμy−jyj.

Initial conditions and the number of variables of the state vector also can be described as the following fuzzy sets:

(9) D−i=xiμD−ixi,S−=SμS−S.

Suppose that the membership functions of the input and output linguistic variables are defined as the following analytical functions:

(10) μX−ixi=φx,aXi,b1X−i,b2X−i,β1X−i,β2X−i=b1X−iaX−i−xβ1X−isign⁡b1X−iaX−i−x+12+b2X−ix−aX−iβ2X−isign⁡b2X−ix−aX−i+12+1−1,

(11) μY−jyj=φy,aYj,b1Y−j,b2Y−j,β1Y−j,β2Y−j=b1Y−jaY−j−yβ1Y−jsign⁡b1Y−jaY−j−y+12+b2Y−jy−aY−jβ2Y−jsign⁡b2Y−jy−aY−j+12+1−1.

In (10) and (11), the coefficients $a_{{\overset{-}{X}}_{i}}$, $a_{{\overset{-}{Y}}_{j}}$, $b_{1{\overset{-}{X}}_{i}}$, $b_{1{\overset{-}{Y}}_{j}}$, $b_{2{\overset{-}{X}}_{i}}$, $b_{2{\overset{-}{Y}}_{j}}$, $\beta_{1{\overset{-}{X}}_{i}}$, $\beta_{1{\overset{-}{Y}}_{j}}$, $\beta_{2{\overset{-}{X}}_{i}}$, and $\beta_{2{\overset{-}{Y}}_{j}}$ are mood, width, and slope of the membership functions of the input and output linguistic variables. These coefficients allow forming high variety of shapers of membership functions. They can also act as indicators of information fuzziness for a formal model of the control objects, which are provided as state space equation (2).

We assume that the indicators of quality of the control system (e.g., time of transient process, overshoot, and bug tracking) are given as the following utility functions generated by experts,

(12) Q−=q1μQ−q1,q2μQ−q2,…,qkμQ−qk,μQ−qk=φqk,aQ−k,b1Q−k,b2Q−k,β1Q−k,β2Q−k,

under certain limitations for variables' state space and control signal:

(13) $$\begin{array}{c} g_{1}\left(\;\overset{-}{x},u,\gamma ,t\;\right)<x_{1max},\\ g_{2}\left(\;\overset{-}{x},u,\gamma ,t\;\right)>x_{2min},\\ \vdots \\ g_{2n-1}\left(\;\overset{-}{x},u,\gamma ,t\;\right)<x_{nmax},\\ g_{2n}\left(\;\overset{-}{x},u,\gamma ,t\;\right)>x_{nmin},\\ g_{m-1}\left(\;\overset{-}{x},u,\gamma ,t\;\right)<u_{max},\\ g_{m}\left(\;\overset{-}{x},u,\gamma ,t\;\right)>u_{min}, \end{array}$$

where *φ* is membership function of the quality index of the control system, provided as (6). Then, the problem of the synthesis of control system, for control objects provided as in a fuzzy state equation (2), with the quality evaluation indicator (12) and the system of constraints (13), can be formulated as described below.

First, it is necessary to synthesize control systems with embedded adaptive adjustment parameters of the controller for object controls (2). All signals should be limited in (13) and the transient processes in the system have to satisfy the predetermined quality parameters (12).

For a given statement of the problem, gradient speed search algorithm in the parametric form, with a proportional-integral controller and circuit bootstrapping can be selected [2, 3]. Among available methods of adaptive control with robust characteristics, the gradient speed search algorithm is the least complex. It also fits the constraints on the control signal and its velocity. Then, the control signal is generated based on the fuzzy set values of state space parameters, according to the following modified control signal:

(14) ut+1=kut·umt+∑i=1nkxi∑ t·xi∑ t,

(15) kx1∑ t+1=kx1∑ t1−ωγ3+ωγ5−γ4δ∗tx1∑ t−ωγ5δ∗t+1x1∑ t+1,kx2∑ t+1=kx2∑ t1−ωγ3+ωγ5−γ4δ∗tx2∑ t−ωγ5δ∗t+1x2∑ t+1,⋮kxn∑ t+1=kxn∑ t1−ωγ3+ωγ5−γ4δ∗txn∑ t−ωγ5δ∗t+1xn∑ t+1,kut+1=kut1−ωγ1+ωγ6−γ2δ∗tumt−ωγ6δ∗t+1umt+1,

where *t* = *mω*, *ω* > 0 is discretization step, *γ* = {*γ*_1_, *γ*_2_, *γ*_3_, *γ*_4_, *γ*_5_, *γ*_6_} are the parameters of the adaptive controller; $e_{i}^{\sum \;}=\int_{x_{i}}^{}(x_{i}-x_{im})\mu_{{\overset{-}{e}}_{i}}(e_{i})dx_{i}$ is a signal mismatch between the actual parameters of the state vector and desirable model parameters; $\mu_{{\overset{-}{e}}_{i}}(e_{i})=\varphi (e_{i},a_{{\overset{-}{e}}_{i}},b_{1{\overset{-}{e}}_{i}},b_{2{\overset{-}{e}}_{i}},\nu_{1{\overset{-}{e}}_{i}},\nu_{2{\overset{-}{e}}_{i}})$ is the membership function of the signal mismatch, provided as (10); $a_{{\overset{-}{e}}_{i}}=a_{{\overset{-}{x}}_{i}}-x_{im}$, $\nu_{1{\overset{-}{e}}_{i}}=\nu_{1{\overset{-}{x}}_{i}}$, $\nu_{2{\overset{-}{e}}_{i}}=\nu_{2{\overset{-}{x}}_{i}}$, $b_{1{\overset{-}{e}}_{i}}=b_{1{\overset{-}{x}}_{i}}$, $b_{2{\overset{-}{e}}_{i}}=b_{2{\overset{-}{x}}_{i}}$; *x*_*i*_^∑ ^ = ∫_*x*_*i*__ *x*_*i*_ *dx*_*i*_ are the integrated parameters of the state vector; *δ* ^*∗*^[*t*] = ∑_*i*=1_^*n*^ *h*_*i*_ · *e*_*i*_^∑ ^, *h*_*i*_ are the coefficients, obtained by Lyapunov's decision equation and the matrix equation with reference model *B*_M_.

The synthesis of the control law based on fuzzy model can increase the robustness of the gradient speed search algorithm; it can also maintain limitations of the phase trajectories in certain areas under uncompensated information fuzziness [11, 12].

Quality indicators of the control systems are based not only on the best possible dynamic characteristics of object controlling $a_{{\overset{-}{x}}_{i}}(t),a_{{\overset{-}{y}}_{i}}(t)$, but also on the parameters of its fuzziness $a_{{\overset{-}{X}}_{i}}$, $a_{{\overset{-}{Y}}_{j}}$, $b_{1{\overset{-}{X}}_{i}}$, $b_{1{\overset{-}{Y}}_{j}}$, $b_{2{\overset{-}{X}}_{i}}$, $b_{2{\overset{-}{Y}}_{j}}$, $\beta_{1{\overset{-}{X}}_{i}}$, $\beta_{1{\overset{-}{Y}}_{j}}$, $\beta_{2{\overset{-}{X}}_{i}}$, and $\beta_{2{\overset{-}{Y}}_{j}}$, provided as (10) and (11).

Reducing the information fuzziness and, consequently, improving the quality control can be achieved by optimizing the parameters of the membership functions of the input and output linguistic variables and fuzzy-logic controllers. This is the core idea of the algorithm for traffic control systems in crossroads, presented in this study.

## 3. The Concept of the Problem Synthesis Adaptive Fuzzy-Logic Traffic Control Systems

Let us consider the problem of synthesis of fuzzy control system of road traffic under conditions of intense traffic in more detail.

We suppose that there is a crossroad regulated with an equal number of intersecting lanes and well-known traffic intensity of *λ*_1_, *λ*_2_, *λ*_3_, *λ*_4_. The directions of traffic 1 and 3 are perpendicular to the directions 2 and 4.

We introduce the following terms: Δ*T*_1_^*u*^ is the duration of the green signal of the traffic light to the first direction of the traffic *λ*_1_, *λ*_3_; Δ*T*_2_^*u*^ is the duration of the green signal of the traffic light to the opposite direction of the traffic *λ*_2_, *λ*_4_.

Taking into consideration the principle of progressive correction, the control object can be defined as (2). The control process assumes that there is a target goal and the control system functions to achieve it. The quality of functioning (criterion of efficiency) of the control system (12) assumes a degree of adaptability to fulfill its task—ensuring a safe traffic with a minimum delay. Therefore, the problem of optimal traffic control at crossroads can be defined as follows.

It is necessary to identify control signals Δ*T*_1_^*u*^ and Δ*T*_2_^*u*^ (the duration of the green signal of the traffic light for each lane) so that the actual state of the traffic *y*(*t*) was as close as possible to the desired state, when the control signals have certain limitations and the system exposed uncontrolled external and parametric impacts *y* ^*∗*^(*t*).

That is, it is necessary to find such parameters of the traffic control system that the total delay at the crossroads is minimal [12, 13]:

(16) Jt=∫t0ty∗τ,xτ,uτ−yτ,xτ,uτdτ⟶min

or
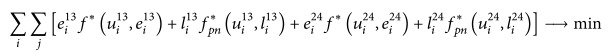
(17)under the constraints

(18) ei13=qileaving13−qiarriv13≤0,ei24=qileaving24−qiarriv24≤0;umin13≤ui13≤umax13,umin24≤ui24≤umax24;0≤li13≤Lmax13,0≤li24≤Lmax24,

where *f* ^*∗*^(*u*_*i*_^13^, *λ*_*i*_^13^) and *f*_*pn*_^*∗*^(*u*_*i*_^13^, *l*_*i*_^13^) are the delay evaluation function and penalty function for the length of queue in front of the stop line of the traffic light in directions 1 and 3, respectively; *f* ^*∗*^(*u*_*i*_^24^, *λ*_*i*_^24^) and *f*_*pn*_^*∗*^(*u*_*i*_^24^, *l*_*i*_^24^) are the delay evaluation function and penalty function for the length of queue in front of the stop line of the traffic light in directions 2 and 4, respectively; *u*_*i*_ is the duration of green signal of traffic light in the* i-stage*; *q*_*i*_^left(13)^, *q*_*i*_^arriv(13)^, *q*_*i*_^left(24)^, and *q*_*i*_^arriv(24)^ are the number of vehicles entered and left the control zone at the crossroad in the *i*th phase, respectively; *u*_min_ and *u*_max_ are the minimum and maximum duration of the green signal of the traffic light, respectively; *l*_*i*_^13^ and *l*_*i*_^24^ are the length of the queue in front of the stop line at directions 1 and 3 and 2 and 4 in the *i*th stage, respectively; *L*_max_ is the maximum allowed length of the queue in front of the stop line.

**Figure 1:**
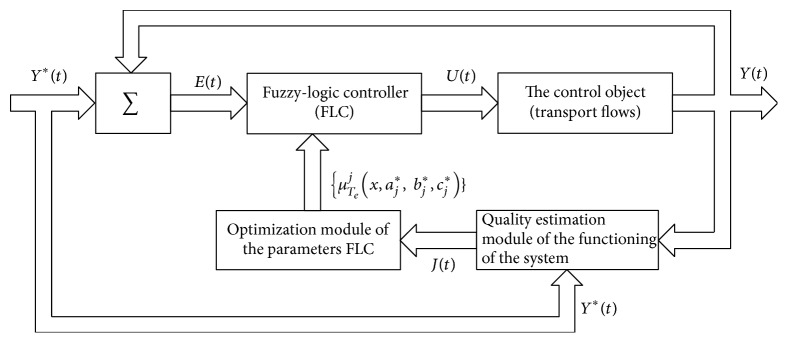
A traffic control system at the crossroads with an adaptive fuzzy-logic controller.

In general, the process control system at the T-shaped crossroads can be rearranged as a feedback control system with adaptive fuzzy-logic controller (Figure 1). In the proposed scheme, the input vector *E* ^*∗*^ = (*e*_1_^*∗*^, *e*_2_^*∗*^) is converted into fuzzy-logic controller (FLC) through fuzzification block. Next, fuzzy inference is performed based on rule base, which results in a fuzzy output variable *u* ^*∗*^. The output values from the FLC are then transferred from the fuzzy region *u* ^*∗*^ to accurate region *u* through defuzzification block [14, 15].

In general, rule bases or knowledge bases contain descriptions of the linguistic variables of the FLC that are predefined for each of the input and output variables. Therefore, we introduce the following linguistic variables:
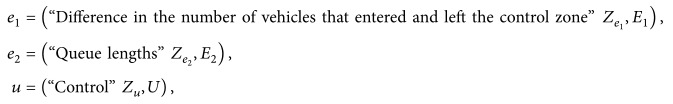
(19)where *Z*_*e*_*i*__ = {*Z*_*e*_*i*__^1^, *Z*_*e*_*i*__^2^,…, *Z*_*e*_*i*__^*k*^}, *i* = 1,2, and *Z*_*u*_ = {*Z*_*u*_^1^, *Z*_*u*_^2^,…, *Z*_*u*_^*k*^}, are the term-sets of linguistic variables *e*_1_, *e*_2_, and *u* with corresponding membership functions (MF) *Z*_*e*_*i*__^*l*^ = *μ*_*e*_*i*__^*l*^(*e*_*i*_), $Z_{u}^{l}=\mu_{l}^{}(u),\;\;l=\bar{1,k}$, given by universal sets *E*_*i*_ = [*E*_*i* min_, *E*_*i* max_] and *U* = [*U*_min_, *U*_max_].

We assume that seven terms *Z*_*x*_ = {“NB”, “NM”, “NS”, “ZE”, “PS”, “PM”, “PB”} correspond to each of the input and output linguistic variables *Z*_*x*_ = {*T*_*e*_1__, *T*_*e*_2__, *T*_*u*_} with triangular membership functions, provided as (10). As a result, the following linguistic variables are derived from the fuzzification processes: 
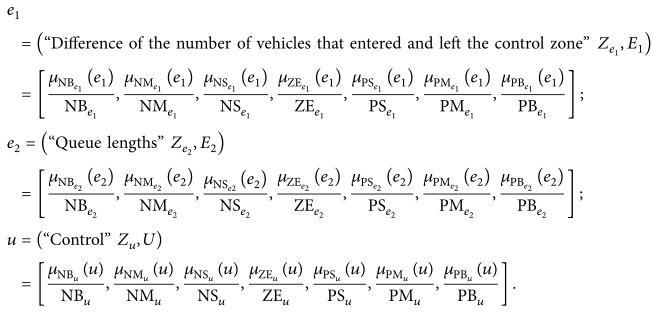
(20)


Next, we develop a rule base for the FLC inferences as follows:

(21) IF Ze1j×Ze2jELSE Zuj,j=1,7,

where (*Z*_*e*_1__^*j*^ × *Z*_*e*_2__^*j*^) is the Cartesian product of the fuzzy sets *E*_1_ and *E*_2_, with the following membership function:

(22) $$\begin{array}{c} \mu_{\left(Z_{e_{1}}^{j}\times Z_{e_{2}}^{j}\right)}\left(\;e_{1},e_{2}\;\right)=\mu_{Z_{e_{1}}^{j}}\left(\;e_{1}\;\right)\wedge \mu_{Z_{e_{2}}^{j}}\left(\;e_{2}\;\right). \end{array}$$

The corresponding output fuzzy set is defined by fuzzy relation $R^{j}=(Z_{e_{1}}^{j}\times Z_{e_{2}}^{j})\times Z_{u}^{j},\;\;j=\bar{1,7}$, with the following membership function:

(23) μRje1,e2,u∗=μZe1je1∧μZe2je2∧μZuju∗.

The set of all rules *R* = ⋃_*j*=1_^7^ *R* ^*j*^ with fuzzy membership function:

(24) μRe1,e2,u∗=⋁j=17μZe1je1∧μZe2je2∧μZuju∗

which defines the rule base of the FLC and produces an algorithm of fuzzy control system.

As a result, for given values of the input linguistic variables *Z*_*e*_1__^*j*^ and *Z*_*e*_2__^*j*^, the fuzzy output value of the logical controller *Z*_*u*_^*j*^ can be determined based on the following composite rule [12]:

(25) $$\begin{array}{c} B^{j}=\left(\;Z_{e_{1}}^{j}\times Z_{e_{2}}^{j}\;\right)\cdot R \end{array}$$

with a degree of membership

(26) μZuju∗=⋁e1∈E1, e2∈E2μZe1je1∧μZe2je2∧μRe1,e2,u∗.

When the linguistic input variables *e*_1_ and *e*_2_ correspond to fuzzy sets *Z*_*e*_1__′ and *Z*_*e*_2__′, fuzzy set *Z*_*u*_′ of the linguistic variable of the control signal *u* ^*∗*^ is determined as follows:

(27) μTu′′u∗=maxe1,e2⁡∏i=1nμTei′ei·minj=1m∏i=1nμTeijei·μTuju∗.

In order to obtain a true output value following fuzzy computations, it is necessary to perform defuzzification—the transformation of fuzzy linguistic variable value (qualitative) *u* ^*∗*^ into discrete numerical value of *u*. For this purpose, center of gravity method is applied [12]:

(28) $$\begin{array}{c} u=\frac{\sum_{n=1}^{7}u_{n}^{*}\mu_{Z_{u}}\left(u_{n}^{*}\right)}{\sum_{n=1}^{7}\mu_{Z_{u}}\left(u_{n}^{*}\right)}. \end{array}$$

Membership function of fuzzy values *Z*_*u*_′ can be represented as

(29) $$\begin{array}{c} \mu_{{Z_{u}^{\prime }}^{\prime }}\left(\;u\;\right)=\left\{\begin{array}{cc} \overset{n}{\underset{i=1}{\prod }}\mu_{Z_{e_{i}}^{j}}\left(e_{i}\right), & u=\varphi^{j},\\ 0, & u\neq \varphi^{j}, \end{array}\right. \end{array}$$

where *φ* ^*j*^ is the discrete numerical value of the output signal.

Then, the final output value of the FLC at the defuzzification stage can be computed as follows:

(30) u=∑j=1mφj∏i=1nμZeijei∑j=1m∏i=1nμZeijei,or  ue−,φ−=∑j=1mφjψje−,

where

(31) $$\begin{array}{c} \psi_{j}\left(\;\overset{-}{e}\;\right)=\frac{\prod_{i=1}^{n}\mu_{T_{e_{i}}^{j}}\left(e_{i}\right)}{\sum_{j=1}^{m}\prod_{i=1}^{n}\mu_{T_{e_{i}}^{j}}\left(e_{i}\right)}. \end{array}$$

If we consider that the basic equation of the road traffic controller can be written as [12]

(32) $$\begin{array}{c} u\left(\;t\;\right)=u_{0}+K\left(\;e\left(\;t\;\right)+\frac{1}{T_{u}}\overset{t}{\underset{0}{\int }}e\left(\;\tau \;\right)d\tau +T_{d}\frac{de\left(t\right)}{dt}\;\right), \end{array}$$

then the rule of the fuzzy traffic control system can be represented as a logic controller with the varying coefficient, as in (14):

(33) $$\begin{array}{c} u\left(\;t\;\right)=u_{0}+K^{*}\left(\;\alpha ,\beta \;\right)\circ \psi_{u_{0}}^{*}{\left(\;\overset{-}{e}\;\right)}^{*}, \end{array}$$

where *K* ^*∗*^(*α*, *β*) is the variable part of the gain that depends on the current dynamic parameters of the road traffic [16]. This allows implementing an adaptive traffic control system as a fuzzy system consisting of two interconnected modules: “module of fuzzy controller of the switching phase traffic lights” and “module of fuzzy adaptive correction of the parameters of the main fuzzy controller of the switching phase traffic lights” *α* and *γ*.

Furthermore, we introduce the following linguistic variables to assess the quality of the control system and adaptive fuzzy correction of the parameters of the fuzzy-logic controller:

(34) de2=“The  Derivative  Queue  Lengths  of  Vehicles”  Zde2,Ede2,Q=“The  Quality  Control  System”  ZQ,EQ,

where *Z*_*de*_2_*i*___ = {*Z*_*de*_2__^(1)^, *Z*_*de*_2__^(2)^,…, *Z*_*de*_2__^(*n*)^}, $Z_{{\overset{-}{Q}}_{i}}=\{{Z_{\overset{-}{Q}}^{\left(1\right)}}_{e_{i}},Z_{\overset{-}{Q}}^{\left(2\right)},\ldots ,Z_{\overset{-}{Q}}^{\left(k\right)}\}$ t are the term-sets of linguistic variables *de*_2_ and *Q* with corresponding membership functions:

(35) Zde2=de2iμZde2de2i,i=1,n¯,ZQ−=qjμQ−qj,j=1,k¯,

given by universal sets *E*_*de*_2__ = [*E*_*de*_2_min_, *E*_*de*_2_max_] and *E*_*Q*_ = [*E*_*Q*__min_, *E*_*Q*__max_].

Then, according to (12) and (25), the choice of the optimal adjustment parameters of the FLC can be represented as the following fuzzy relations:

(36) $$\begin{array}{c} R_{Q^{*}}^{j}=\left(\;Z_{e_{2}}^{j}\times Z_{e_{2}}^{j}\;\right)\times Z_{Q}^{j},\;j=\bar{1,N_{Q}}, \end{array}$$

where

(37) Z−Q=Q1TPμQ−1TPQ1TP,μQ−kTPQkTP=φQkTP,aQ−k∗,b1Q−k∗,b2Q−k∗,β1Q−k∗,β2Q−k∗,φQkTP,aQ−k∗,b1Q−k∗,b2Q−k∗,β1Q−k∗,β2Q−k∗=b1Q−k∗aQ−k∗−xβ1Q−k∗·sign⁡b1Q−k∗aQ−k∗−x+12+b2Q−k∗x−aQ−k∗β2Q−k∗sign⁡b2Q−k∗x−aQ−k∗+12+1−1,Q−=q1μQ−q1,q2μQ−q2,…,qkμQ−qk,μQ−qk=φqk,aQ−k,b1Q−k,b2Q−k,β1Q−k,β2Q−k.

**Figure 2:**
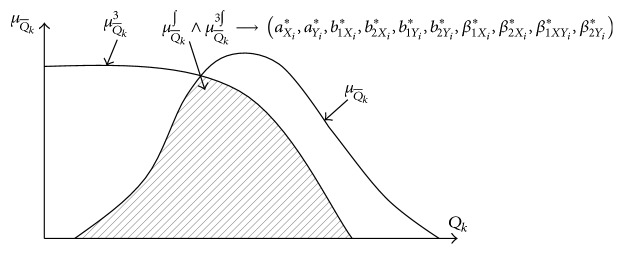
Graphic illustration of a choice of optimum adjusting parameters.

This fuzzy relationship served as the basis for developing algorithms of adaptive parameter settings of the fuzzy-logic controller (Figure 2).

## 4. Simulation Modeling and Analysis of Fuzzy Traffic Control System

We develop a simulation model of the adaptive fuzzy control system of road traffic on Matlab software package. The simulation model is used to test the efficiency of the fuzzy control system through series of computational experiments.

As an object of simulation, we chose one of the heavy-loaded and problematic crossroads in Tashkent city, Uzbekistan. The simulation model considered the road traffic intensity, observed at the crossroad during the peak-hours from 07:00 to 10:00 on May 5, 2015. The experiment consisted of two phases. At each phase of the simulation, various patterns of traffic-light management were used. In the first phase, the traffic lights with fixed cycles, which are used in real life, are simulated. In the second phase, traffic lights controlled with adaptive fuzzy system were simulated. For this purpose, the rules of fuzzy control system with a module for adaptive adjustment of parameters were synthesized using the proposed method.

**Figure 3:**
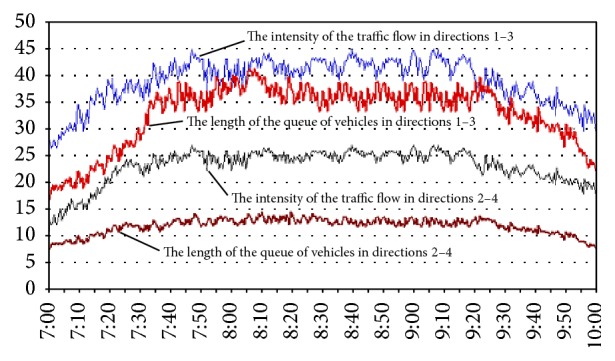
The results of the simulation modeling of the traffic control system with optimum fixed cycle of traffic lights.

**Figure 4:**
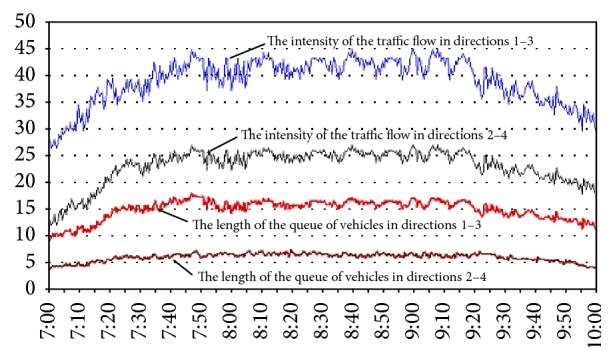
The results of the simulation modeling of the adaptive traffic control system.

The simulation results are presented below.  Figure 3 shows the results of simulation of the road intersection that is managed by traffic lights with fixed cycles.  Figure 4 shows the results of simulation of the crossroad that is managed by adaptive fuzzy controller.

Simulation results show that the synthesized fuzzy control system successfully solved the problem of queues at traffic lights and performed more effective than the current traffic control system with fixed-cycle traffic lights. Particularly, the length of queue for the synthesized fuzzy control system is less than 2.6 times for traffic control system with fixed-cycle traffic lights. Also, delay time for synthesized fuzzy control system is 27% less than traffic control system with fixed-cycle traffic lights.

Based on the results of computational experiments, we can conclude that the synthesized adaptive fuzzy control system of road traffic in conditions of intensive traffic is robust and allows controlling road traffic within a wide range of parameters.

## 5. Conclusion

In this study, we formally described the problems of synthesis of adaptive fuzzy traffic control systems in conditions of intense road traffic. We developed efficient algorithms of the synthesis of the adaptive fuzzy-logic traffic control systems. We also developed a simulation model of adaptive fuzzy-logic control system for managing road traffic and conducted a series of computational experiments.

The results showed that the synthesized fuzzy-logic traffic control system, based on the proposed method, allows controlling crossroads more effectively compared to traffic control system with fixed-cycle traffic lights. We conclude that adaptive fuzzy-logic controller can successfully function in the condition of information fuzziness. It can also successfully function when exposed to uncontrolled external and parametric impacts. The implementation of the solution in heavy-loaded crossroads allows reducing the length of queues at the traffic lights up to 2.6 times and the delay time up to 27%.
